# The effects of lettuce extract on the level of T4, memory and nerve conduction velocity in male rats

**Published:** 2020

**Authors:** Majid Jafari Nejad Bajestani, Mahdi Yousefi, Mousa-Al-Reza Hadjzadeh, Mahmoud Hosseini, Ali Taghipour, Shiba Yousefvand, Hamid Reza Ghorbanzadeh

**Affiliations:** 1 *Department of Persian Medicine, School of Persian and Complementary Medicine, Mashhad University of Medical Science, Mashhad, Iran*; 2 *Department of Physiology, School of Medicine Mashhad University of Medical Sciences, Mashhad, Iran*; 3 *Division of Neurocognitive Sciences, Psychiatry and Behavioural Sciences Research Center, Mashhad University of Medical Sciences, Mashhad, Iran*; 4 *Neurogenic Inflammation Research Center, Mashhad University of Medical Sciences, Mashhad, Iran*; 5 *Department of Epidemiology & Biostatistics, School of Health Social Determinants of Health Research Center, Cancer Research Center Mashhad University of Medical Sciences, Mashhad, Iran*

**Keywords:** Lettuce, T4 level, Coldness, Nerve conduction velocity, Rats, Memory

## Abstract

**Objective:**

According to the traditional medicine, lettuce can affect nerve conduction velocity and memory. So, to investigate the effect of lettuce seeds extract on body activities, lettuce seeds were used.

**Materials and Methods:**

In the present study, the effects of lettuce (*Lactuca sativa*) seeds extract consumption (in drinking water) on T4 level, animals' weight, water and food consumption, nerve conduction velocity (NCV), and memory in Wistar rats, were investigated. In this study, 24 Wistar rats were used, and divided into three groups: control, L 200 mg/kg, and L 400 mg/kg.

**Results:**

The results showed that, the T4 level, food and water intake, time spent and distance travelled in Q1, delay time to enter and the number of entrance into the dark room in both treated groups were not significantly different from the control group. Animal weight and NCV, in 400 mg/kg group were not significantly different from the control group, but in 200 mg/kg group, they were significantly decreased (p<0.05). The duration spent in the dark room (48 hr after shock) in L 400 mg/kg increased compared to the control group (p<0.05), but in L 200 mg/kg group at all time points, and in L 400 mg/kg treated group 3 and 24 hr after shock, it was not significantly different from the control group.

**Conclusion:**

Based on these findings, the T4 level, memory, food and water intake were not changed by lettuce extract, while NCV and animal weight were decreased following treatment with lettuce extract.

## Introduction

The scientific name of lettuce is *Lactuca saliva*. Lettuce is a plant from the *Compositae* family that contains several compounds, including: lactocin, maleic acid, asparagine, oxalic acid, and succinic acid. According to the traditional medicine, lettuce seeds have a cold and dry nature (Momen Tonekaboni et al., 2008 ▶). Lettuce seeds extract has analgesic and anti-inflammatory effects (Sayyah et al., 2004 ▶). Lettuce extract inhibits scopolamine induced- amnesia in mice (Malik et al., 2018 ▶). Consumption of high doses of lettuce extract can lead to muscle flaccid paralysis. It was argued that this effect is due to the impact of lettuce extract on nerve conduction velocity (Gonzálex-Lima et al., 1986 ▶). Excessive consumption of lettuce causes forgetfulness (Momen Tonekaboni et al., 2008 ▶; Choopani et al., 2015 ▶).

Among the various physiological systems in the body, the thyroid gland has a central role in the development and differentiation of cells during embryonic and neonatal period and adulthood (Goncalves et al., 2006 ▶; Farahani et al., 2013 ▶; Karbalaei et al., 2013 ▶). Thyroid hormones by affecting cellular metabolism, affect body tissues (Harvey and Williams, 2002; Lin et al., 2005 ▶). It was shown that due to the importance of the role of thyroid hormones in the central nervous system, thyroid hormone abnormalities can cause dementia and memory impairment by affecting basal metabolism (Leach et al., 2015 ▶; Pasand et al., 2016 ▶).

According to the above-noted information, lettuce seeds can affect nerve conduction velocity and memory. As well, combinations with cold nature could affect T4 level. Therefore, the aim of this study was to investigate the effect of induction of experimental coldness in body with consumption of lettuce seeds extract on memory, nerve conduction velocity, T4 level, food intake, and water intake and body weight in male rats.

## Materials and Methods

**Animals**

In this study, 24 male Wistar rats with an average weight of 180-220 g, were used. The rats were divided into three groups randomly, and kept and treated in accordance with the ethical protocols of laboratory animals approved by Mashhad University of Medical Sciences (Ethics approval No. IR MUMS. REC 1395.575). The rats were kept and treated under standard conditions (at 20-24°C, with 12 hr of dark/light cycle), and they had free access to food (Javaneh Khorasan, Iran), and tap drinking water. The body weight of rats and their 24-hr water and food intake (one day in a week) were measured and recorded. In this study, the control group received ordinary tap drinking water for 6 weeks. The second group (L 200) received water-soluble extract of lettuce seeds at a dose of 200 mg/kg/day, and the third group (L400) received water-soluble extract of lettuce seeds at a dose of 400 mg/kg/day for 6 weeks. Doses were determined based on previous studies (Harsha et al., 2013 ▶; Ahangarpour et al., 2014 ▶; Ghorbani et al., 2013 ▶).

**Preparation of extracts**

In this study, 970 g of lettuce seeds was purchased from a local market in Mashhad, and used for all experiments. Lettuce seeds were powdered by a grinder, then, the powder was soaked in 70% ethanol for 72 hr at 45-40°C, and this solution was shacked for several hours. After that, this solution was filtered through different filters and finally, filtered by a vacuum pump and strainer, therefore, the pulp was removed. The extract was dried in a vacuum oven at 40°C. Finally, 105.6 g of lettuce seeds extract was obtained, and stored at 4°C until use.

**Measurement of spatial learning and memory**

Morris Water Maze has been widely used to investigate certain aspects of spatial memory. Morris Water Maze is a water tank with a diameter of 136 cm, a height of 60 cm, and a depth of 30 cm; almost half of that height is filled with water (20-24°C). The surface of the maze is imaginary divided into four equal quarter and a circular platform (28 cm height, with 10 cm diameter), is hidden 2 cm below the water level. It is placed in the center of the southwest quarter (the target quarter). The maze is located in a room, where there are several different spatial symptoms, and these symptoms are constant during the experiments and visible to the animals in the maze. This collection is monitored through a detector camera positioned at a height of 180 cm above the center of the water maze, and connected to a computer. Data is collected and stored while the experiments are being done. Before the experiment, each animal was released from one of the four position (North (N), East (E), South (S), and West (W)) of the maze. The choice of location was started randomly by experimenter (Murphy 2013 ▶). Each animal had a maximum period of 60 sec at each test time to find and stand on the platform using fixed signs on the wall, and stay 15 sec on it. If the animal was not able to find the platform within 60 sec, it was placed on the platform by the experimenter. Time spent to find the platform, and the distance travelled to find the platform were recorded by the camera. Rats had four training days, and on the 5th day, spatial memory test was carried out. The platform was removed, and the rats were swimming for 60 sec. The time spent in the target quarter (Q1), and the distance travelled in that quarter in comparison with other quarters were calculated.

**Passive avoidance test**

Passive avoidance test is a classical test designed to evaluate the non-linear (conceptual) memory communication in small laboratory animals. The passive avoidance phrase is usually used to explain the experiments in which, animals learn to avoid a painful stimulus. The shuttle box is used for passive avoidance test. The shuttle box is a box with two rooms, separated from each other by a guillotine door. There is a dark room and a bright room, and the bottom of the box is made of metal bars. The animals training stage was performed three days before the main test. Each animal was placed in the bright room for 20 sec. Then, the door was opened and the animals due to the desire to enter the dark room, entered it. Upon the arrival of the animal into the dark room, an electric shock with a frequency of 100 Hz and a 0.5 miliamper (mA) for 5 sec, was applied via the rods below the animal's feet in the dark room. Next, 24 hr after the last training, the avoidance memory test was performed. On the experiment day, 2 sec after the presence of the animal in the bright room, the guillotine door was opened, and the time of the animal's delay to enter the dark room and the duration of its presence in the dark room, were recorded for 180 sec. During the experiment, no electric shock was applied to the animal in the dark room.

**Nerve conduction velocity measurement**

Measurement of nerve conduction velocity is a major laboratory technique for the study of peripheral nerve function. At the end of the experiment (day 42), the animals were anesthetized with intraperitoneal injection of a combination of xylazine and ketamine (a dose of 80 mg/kg ketamine and 8 mg/kg xylazine) (Alfasan, Netherlands). After ensuring complete anesthesia, the animals were fixed on the laboratory board. To determine the motor nerve conduction velocity, first, the right sciatic nerve of the animal was stimulated by 10V via inserting a needle-induced stimulation electrode in the sciatic hole. Then, the tibial nerve was stimulated in the animal's knee. To record the motor response, needle surface stability electrode was placed in the animal's claws. Sciatic-tibial motor neural conduction velocity using two stimulation point along the nerve, was calculated. (Etxeberria et al., 2007 ▶).

**The measurement of T4 level**

T4 level was measured using a radioimmunoassay kit (Poua Patan Goster Co, Iran), and read by a gamma counter.

**Estimation of food and water consumption**

Food and water intake by rats were measured for 24 hr every week during the experiment. Animals were also weighed weekly.

**Data analysis**

The results are expressed as Mean±SEM. Delay time and the distance travelled to find the platform, and the data from passive avoidance test were analyzed by repeated measure analysis of variance (ANOVA). The time spent in the target quarter and the distance travelled in this quarter and other quarters, and the other parameters measured in this sturdy, were analyzed by one way ANOVA, and compared by LSD *post-hoc*. A p<0.05 was considered significant.

## Results

In this study, the effects of lettuce extract on T4 level, animal weight, food and water intake, nerve conduction velocity, and finally, spatial and avoidance memory at the end of 6 week treatment with lettuce seeds extract, were assessed and the results are shown in Figures 1-9.

**Figure 1:**
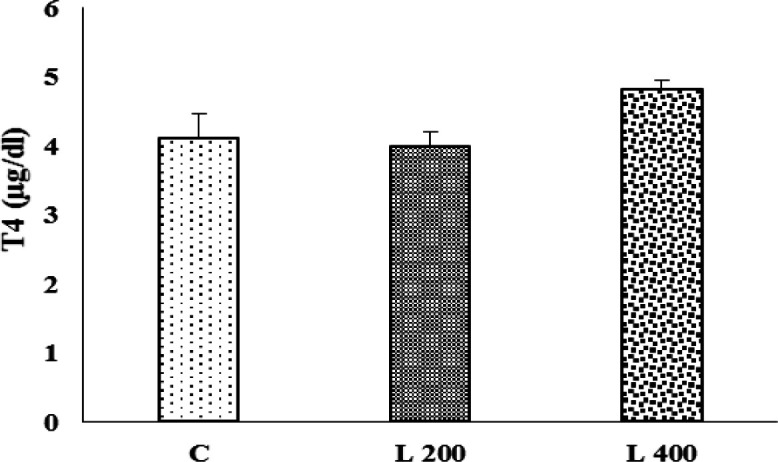
The effect of lettuce seeds extract (L 200 mg/kg and L 400 mg/kg) on the serum T4 level in rats. Data are expressed as Mean±SEM. The data were analyzed by repeated measure analysis of variance (ANOVA). C: control, L: lettuce. (n=6 in each group).

**The effect of lettuce extract on the T4 level**

Figure 1 shows the level of T4 after 42 day treatment with lettuce extract. The results showed that the T4 level in L 200 and 400 mg/kg treated groups, did not change significantly compared to the control group (Figure 1).

**Figure 2:**
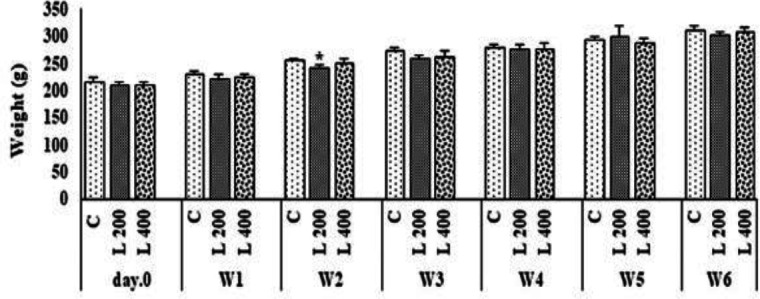
The effect of different doses of lettuce seeds extract on rats’ weight. Data are expressed as Mean±SEM. *p<0.05 compared to the control group. The data were analyzed by repeated measure analysis of variance (ANOVA). W: week, C: control, L: lettuce. (n=6 in each group)

**Figure 3:**
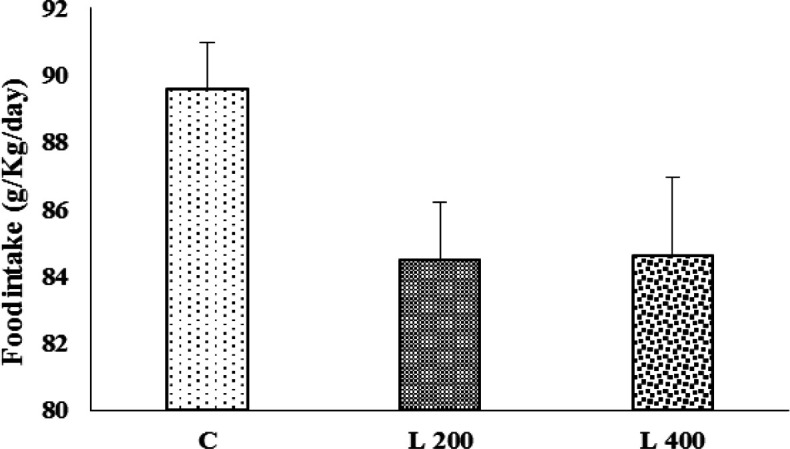
The effect of lettuce seeds extract (L 200 mg/kg and L 400 mg/kg) on food intake in rats. Data are expressed as Mean±SEM. The data were analyzed by repeated measure analysis of variance (ANOVA). C: control, L: lettuce. (n=6 in each group).

**The effect of lettuce extract on body weight, and food and water intake**

**Figure 4:**
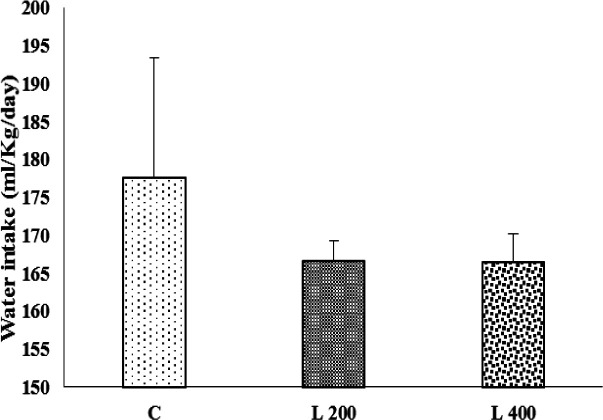
The effect of lettuce seeds extract (L 200 mg/kg and L 400 mg/kg) on water intake in rats. Data are expressed as Mean±SEM. The data were analyzed by repeated measure analysis of variance (ANOVA). C: control, L: lettuce. (n=6 in each group)

Figures 2, 3, and 4 indicate the effect of lettuce extract consumption on body weight, and food and water intake. The results showed that body weight in the 400 mg/kg group, did not change vs. control group, but in 200 mg/kg treated group, significantly decreased when compared to the control group (p<0.05) (Figure 2). The amount of food and water intake in both 200 and 400 mg/kg lettuce groups was not significantly different from the control group (Figures 3 and 4). 

**The effect of lettuce extract on the nerve conduction velocity**

**Figure 5:**
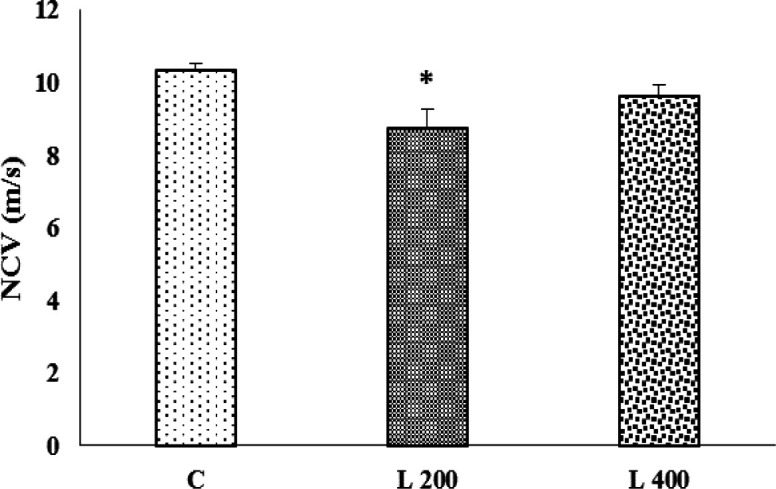
The impact of different doses of lettuce seeds extract (L 200 mg/kg and L 400 mg/kg) on nerve conduction velocity (NCV) in rats. Data are expressed as Mean±SEM. *p<0.05 compared to the control group. The data were analyzed by repeated measure analysis of variance (ANOVA). C: control, L: lettuce. (n=6 in each group).

As Figure 5 shows, nerve conduction velocity in 400 mg/kg treated group, did not change vs. control group, but in 200 mg/kg treated group, significantly decreased when compared to the control group (p<0.05) (Figure 5).

**The effect of lettuce extract on spatial learning and memory**

**Figure 6:**
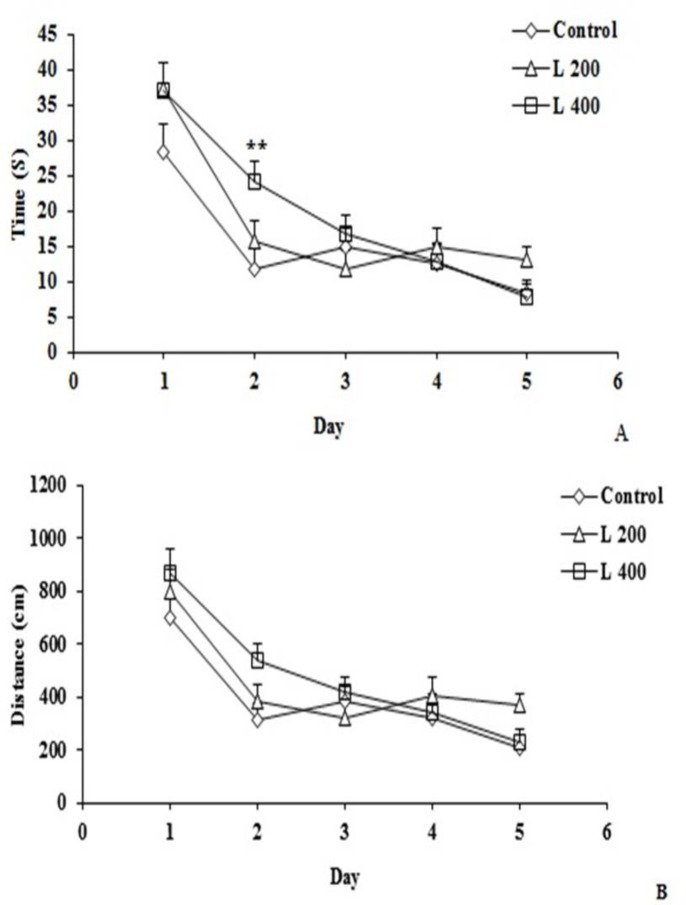
The effect of different doses of lettuce seeds extract (L 200 mg/kg and L 400 mg/kg) on spatial learning and memory in rats. Data are expressed as Mean±SEM. **p<0.01 compared to the control group. The data were analyzed by repeated measure analysis of variance (ANOVA). C: control. L: lettuce. (n=6, in each group)

As Figure 6 demonstrates, the time to find the platform in the 200 mg/kg lettuce extract group and the distance travelled to find the platform in both lettuce treated groups, on all training days did not change significantly compared to the control group, but the time to find the platform in the 400 mg/kg lettuce group on the second day of training significantly decreased when compared with the control group (p<0.01) (Figure 6).

**Figure 7.**
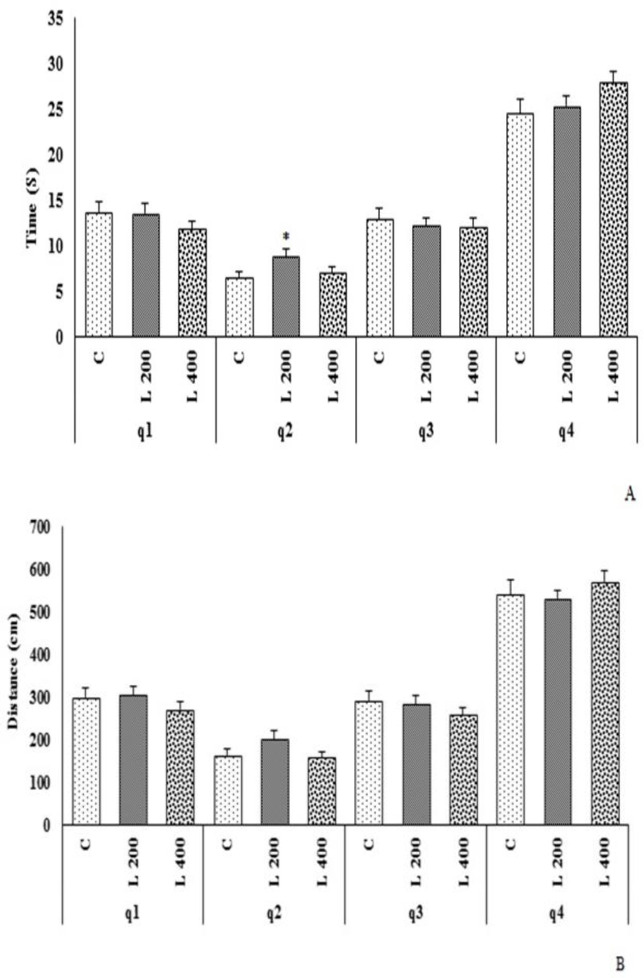
The effect of different doses of lettuce seeds extract (L 200 mg/kg and L 400 mg/kg) on spatial learning and memory in rats. Data are expressed as Mean±SEM. *p<0.05 compared to the control group. The data were analyzed by repeated measure analysis of variance (ANOVA). Q: quarter. C: control. L: lettuce. (n=6 in each group)

As Figure 7 indicates, on the probe day, in both lettuce treated groups (L 200 and L 400 mg/kg), there was no change in the time spent in the target quarter (Q1), and the distance travelled in target quarter (Q1) in compression to the control. In 200 mg/kg lettuce treated group, the time spent in the non-target quarters (Q2 and Q4) significantly increased in comparison to the control group (p<0.05) (Figure 7).

**The effect of lettuce extract on the passive avoidance memory**

**Figure 8:**
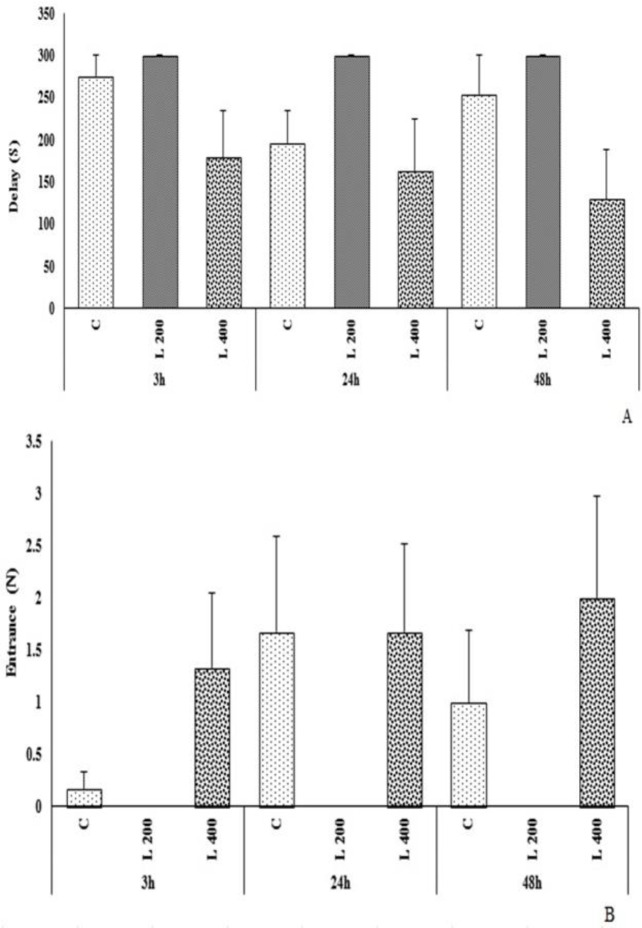
The effect of different doses of lettuce seeds extract (L 200 mg/kg and L 400 mg/kg) on passive avoidance memory in rats. Data are expressed as Mean±SEM. *p<0.05 compared to the control group. The number of entrance to the dark room and the time spent in the dark room in L 200 mg/kg group (3, 24, and 48 hr after shock) was zero (0) (Figure 8 B). The data were analyzed by repeated measure analysis of variance (ANOVA). H: hour. C: control. L: control. (n=6 in each group)

As Figure 8 shows, the delay time to enter the dark room, and the number of entrance to the dark room, in both L 200 and L 400 mg/kg treated groups, were not significantly different from those of the control group (Figure 8).

**Figure 9:**
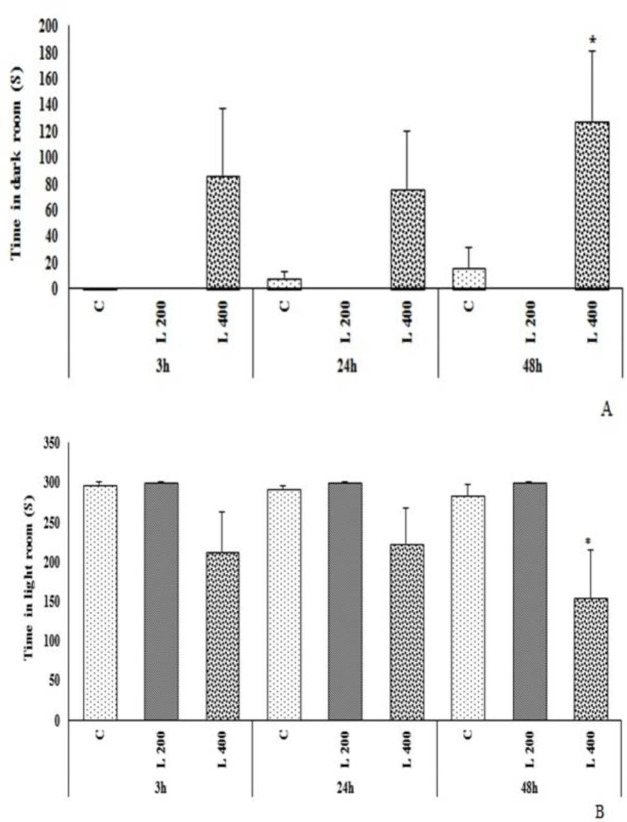
The effect of different doses of lettuce seeds extract (L 200 mg/kg and L 400 mg/kg) on passive avoidance memory in rats. Data are expressed as Mean±SEM. *p<0.05 compared to the control group. The number of entrance to the dark room and the time spent in the dark room in L 200 mg/kg group (3, 24, and 48 hr after shock) was zero (0) (Figures 9 A). The data were analyzed by repeated measure analysis of variance (ANOVA). H: hour. C: control. L: lettuce. (n=6 in each group)

As Figure 9 presents, the duration of stay in the dark room in the L 400 mg/kg group at 48 hours after shock was significantly higher than the control group (p<0.05). The duration of stay in the bright room in the L 400 mg/kg treated group at 48 hours after shock was significantly decreased compared to the control group (p<0.05). The duration of stay in the dark room and bright room in L 200 mg/kg treated group at all times (3, 24 and 48 hr), after shock was not significantly different vs. the control group (p>0.05). The duration of stay in the dark room and bright room in L 400 mg/kg treated group 3 and 24 hr after shock, was not different from the control group (Figure 9).

## Discussion

In the present study, the effects of lettuce seeds extract on serum level of T4, animal weight, water and food intake, memory, and nerve conduction velocity in treated rats, were investigated. The results of this study showed that the serum thyroxin level in rats treated with the ethanoic extract of both doses of lettuce seeds for 6 weeks was not significantly different with control group. 

Although in our study lettuce was not able to impinge upon serum T4 level. The short duration of treatment with lettuce extract maybe a reason in this experiment that lettuce was not able to reduce T4 level.Therefore, lettuce extract could not exert its effect on thyroid gland function, and was not able to reduce T4 level.

In this study, treatment with ethanoic extract of lettuce seeds (in drinking water) had no effect on memory. In line with our study, it was shown that lettuce consumption in drinking water has no effect on memory in Lymnaea (A kind of snail) (Haney and Lukowiak, 2000 ▶). This finding is, in line with our results and confirms our findings. In rats with Alzheimer's disease (amyloid beta-induced Alzheimer's), consumption of alcoholic extract of lettuce leaf for 21 days at 100 and 200 mg/kg doses, reduced memory deficit (Postu et al., 2018 ▶).These findings are inconsistent with our results, because this study investigated the effect of lettuce extract on memory impairment due to Alzheimer's disease, while our study investigated the effect of lettuce extract in rats without memory impairment. In our research, the treatment period was longer and higher doses (200 and 400 mg/kg) were used. These factors may be the reasons for which our results differ from those reported by Postu et al. (2018) ▶. 

In our study, the nerve conduction velocity was decreased by consumption of lettuce seeds extract. Lettuce extract was shown to reduce the nerve conduction velocity which is in line with the results of the present study (Gonzálex-Lima et al., 1986 ▶; Dasgupta et al., 2005 ▶; Avicenna, 2005 ▶; AiA, 2008 ▶).

The results of the current study showed that consumption of lettuce seeds extract reduced animal body weight. Adjrah et al. showed that, lettuce consumption for 28 days led to a significant reduction in body weight in rats; their finding is in favour of our results (Adjrah et al., 2013 ▶). It was reported that consumption of lettuce was had no effect on mouse body weight (Lee et al., 2009 ▶), which is in contrast with our results probably due to differences in race of animals used the treatment period. According to our results, lettuce seeds extract consumption did not affect water intake. 

However, this study had limitations, including, due to the time and cost limitation, there was no possibility to investigate different doses of lettuce seed extract. To the best of our knowledge, as we investigated the literature, few similar studies were found to compare with our study.

In summary, our results showed that treatment with lettuce seeds extract, did not significantly change T4 level serum and memory. This extract at both doses, reduced the nerve conduction velocity. Consumption of ethanolic lettuce seeds extract decreased the body weight, without affecting food and water intake. Perhaps, validity of these results may need more investigations to find out cellular and molecular mechanisms. 
